# Influence of salinity, nitrogen and phosphorus concentrations on the physiological and biochemical characteristics of two Chlorophyceae isolated from Fez freshwater, Morocco

**DOI:** 10.1038/s41598-024-58864-4

**Published:** 2024-04-09

**Authors:** Bouchra Assobhi, Houda Ennasry, Salma Latique, Mohammed Kara, Mohammed Alaoui Mhamdi, Jamila Bahhou

**Affiliations:** 1 Laboratory of Biotechnology, Conservation and Valorization of Natural Resources, Faculty of Sciences Dhar El Mahraz, Sidi Mohamed Ben Abdellah University, Fez, Morocco; 2 Laboratory of Toxicology and Pharmacology, Faculty of Medicine and Pharmacy, Sidi Mohamed Ben Abdellah University, Fez, Morocco

**Keywords:** Carbohydrates, Environmental biotechnology, Lipids, Biofuels, Salt

## Abstract

Microalgae are widely exploited for numerous biotechnology applications, including biofuels. In this context, *Chlamydomonas debaryana* and *Chlorococcum* sp. were isolated from Fez freshwater (Morocco), and their growth and lipid and carbohydrate production were assessed at different concentrations of NaCl, NaNO_3_, and K_2_HPO_4_. The results indicate a small positive variation in growth parameters linked to nutrient enrichment, with no considerable variation in carbohydrate and lipid levels in both algae. Moreover, a negative variation was recorded at increased salinity and nutrient limitation, accompanied by lipid and carbohydrate accumulation. *Chlorococcum* sp. showed better adaptation to salt stress below 200 mM NaCl. Furthermore, its growth and biomass productivity were strongly reduced by nitrogen depletion, and its lipid production reached 47.64% DW at 3.52 mM NaNO_3_. As for *Chlamydomonas debaryana*, a substantial reduction in growth was induced by nutrient depletion, a maximal carbohydrate level was produced at less than 8.82 mM NaNO_3_ (40.59% DW). The effect of phosphorus was less significant. However, a concentration of 0.115 mM K_2_HPO_4_ increased lipid and carbohydrate content without compromising biomass productivity. The results suggest that growing the two Chlorophyceae under these conditions seems interesting for biofuel production, but the loss of biomass requires a more efficient strategy to maximize lipid and carbohydrate accumulation without loss of productivity.

## Introduction

Recently, microalgae have emerged as a promising source of a wide variety of bioproducts. They have unique and much sought-after characteristics that make them good alternatives in many biotechnological fields, such as biofuel production^1^. As a result, the number of studies on microalgae continues to grow, as does the number of algal species cultivated and exploited on both small and large scales^2^. However, the development of the third-generation biofuel sector is still hampered by the high cost of large-scale production. Faced with this challenge and to ensure the competitiveness of microalgae-based biofuels in the international market, the selection of potential microalgae and the optimum physicochemical conditions for cultivation are essential^3^.

Isolating and cultivating new algal species will lead to the selection of lipid-accumulating species with biodiesel production potential or carbohydrate-accumulating species considered as candidates for biomethane, bioethanol, or biohydrogen production. To this end, it is always interesting to isolate indigenous microalgae that are better adapted to local environmental conditions and therefore more suitable for successful large-scale cultivation^4^.

Moreover, the physiological and biochemical characteristics of microalgae can vary according to environmental conditions. Under favorable conditions, lipids and carbohydrates are produced in low quantities in most microalgae. Conversely, levels of these reserve molecules increase under stressful conditions^5,6^. Furthermore, the selection of optimal environmental conditions is of great importance in large-scale cultivation since they influence the competitive capacity of the algae^7,8^. As a result, many studies have focused on the cultivation of microalgae under nutrient stress, in particular the limitation or deprivation of nitrogen and phosphorus. Indeed, this type of stress has been reported to trigger the accumulation of lipids and, to a lesser extent, carbohydrates^9–11^. Other studies have concentrated on the use of salt stress as a strategy for increasing lipid and carbohydrate yields. However, these studies have mainly focused on marine or halotolerant species^12^, neglecting a wide range of freshwater microalgae that may offer considerable potential. In addition, the cultivation of freshwater species on saltwater could reduce their dependence on freshwater, a much sought-after advantage given the current problems of water scarcity and climate change.

Thanks to the diversity of aquatic ecosystems and favorable climatic conditions, microalgae cultivation in Morocco began in 2003, limited to the production of food supplements from spirulina^13^. Subsequently, work focused on the isolation and cultivation of other algal species for numerous biotechnological applications^14–17^. However, further research is required to valorize algal resources and develop the microalgae sector in Morocco.

In this context, the present study aims to (1) isolate *Chlorococcum* sp. and *Chlamydomonas debaryana* from local freshwaters (Fez, Morocco) and (2) study their physiological and biochemical responses to different salinities and concentrations of nitrogen and phosphorus in order to derive information for orienting their metabolism towards lipid or carbohydrate production.

## Results

### Effects of different culture conditions on the growth of *Chlorococcum* sp. and *Chlamydomonsa debaryana*

#### Effect of salinity

**Figure 1:**
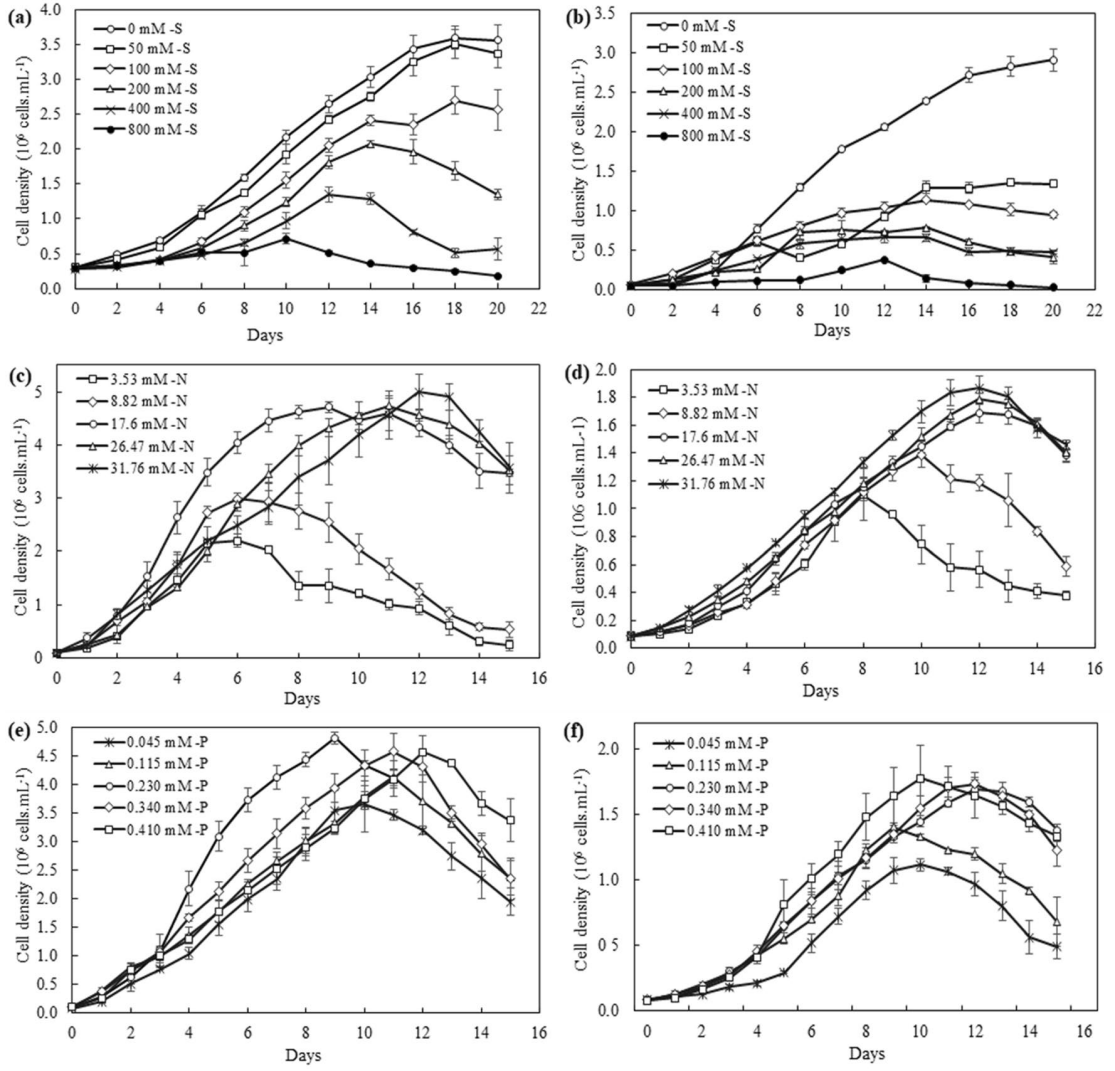
The growth curves of *Chlorococcum* sp. and *Chlamydomonas debaryana* cultivated under different salt (S), nitrate (N), and phosphate (P) concentrations. (**a**) The growth curves of *Chlorococcum* sp. under different salt concentrations (S). (**b**) The growth curves of *Chlamydomonas debaryana* under different salt concentrations (S). (**c**) The growth curves of *Chlorococcum* sp. under different nitrate concentrations (N). (**d**) The growth curves of *Chlamydomonas debryana* under different nitrate concentrations (N). (**e**) The growth curves of *Chlorococcum* sp. under different phosphate concentrations (P). (**f**) The growth curves of *Chlamydomonas debaryana* under different phosphate concentrations (P) (means ± SD).

In contrast to the nitrogen an phosphorus experiments performed over a short period of 15 days, the salinity tests were extended by five days to confirm the tolerance shown by *Chlorococcum* sp. The results obtained show that the control cultures (0 mM NaCl) resulted in maximum growth of the two algae studied, representing a cell density of 3.60 ± 0.17 × 10^6^ cells mL^−1^ for *Chlorococcum* sp. (Fig. 1a) and 2.91 ± 0.14 × 10^6^ cells mL^−1^ for *Chlamydomonas debaryana* (Fig. 1b). In addition, increasing concentrations of NaCl had a different effect on the growth of the two Chlorophyceae. At 50 mM NaCl, *Chlorococcum* sp. showed similar growth dynamic to that observed in the control and a high growth rate of 0.157 d^−1^ (Table 1). This can be explained by the maintenance of photosynthetic activity in the cells, with a high chlorophyll a content (39.030 µg mL^−1^). At 100 and 200 mM NaCl, this microalga showed a longer lag phase, reflecting acclimatization difficulties, and an early decline with a growth inhibitory rate of 32% (Table 1). These findings demonstrate the microalgae's adaptation to moderate salt stress. For *Chlamydomonas debaryana*, the same NaCl concentrations resulted in a greater reduction in cell growth, from 2.91 ± 0.14 × 10^6^ cells mL^−1^ in the control to 1.34 ± 0.02 × 10^6^ cells mL^−1^, corresponding to an inhibitory rate of over 45% (Table 1). Growth rate was also affected, decreasing from 0.278 d^−1^ (0 mM NaCl) to 0.179 d^−1^ (400 mM NaCl) (*P *˂ 0.0001). However, very high salt stress (800 mM NaCl) resulted in strong growth inhibition for the two algae studied (88.359% for *Chlorococcum* sp. and 93.967% for *Chlamydomonas debaryana*) and a reduction in growth rates to 0.012 d^−1^ for *Chlorococcum* sp. and 0.067 d^−1^ for *Chlamydomonas debaryana*. Chlorophyll a content was significantly reduced at 3.038 µg mL^−1^ for *Chlorococcum* sp. and 0.053 µg mL^−1^ for *Chlamydomonas debaryana* (*P* ˂ 0.0001).

**Table 1:** Chlorophyll a content, specific growth rate, and inhibitory rate of *Chlorococcum* sp. and *Chlamydomonas debaryana* at different culture conditions.

	Chl. a (µg mL^−1^)	Growth rate (d^−1^)	Inhibitory rate (%)
NaCl concentrations (mM)			
*Chlorococcum* sp.			
0 (control)	39.030 ± 6.833^a^	0.166 ± 0.004^a^	0.000
50	39.770 ± 7.333^a^	0.157 ± 0.003^ab^	9.326
100	36.423 ± 2.222^a^	0.154 ± 0.003^b^	20.587
200	25.827 ± 4.431^b^	0.139 ± 0.002^c^	31.641
400	9.280 ± 2.767^c^	0.109 ± 0.004^d^	57.616
800	3.038 ± 0.012^d^	0.012 ± 0.005^e^	88.359
*Chlamydomonas debaryana*			
0 (control)	32.217 ± 3.902^a^	0.272 ± 0.001^a^	0.000
50	25.420 ± 1.433^ab^	0.228 ± 0.004^b^	45.876
100	22.223 ± 4.062^ab^	0.202 ± 0.0004^c^	52.519
200	12.310 ± 0.660^b^	0.192 ± 0.002^c^	67.202
400	5.927 ± 0.656^bc^	0.179 ± 0.005^d^	72.408
800	1.053 ± 0.015^d^	0.067 ± 0.020^e^	93.967
NaNO_3_ concentrations (mM)			
*Chlorococcum* sp.			
3.52 (N/P ratio ≈ 15)	3.100 ± 0.533^d^	0.079 ± 0.013^c^	91.157
8.82 (N/P ratio ≈ 38)	3.133 ± 0.156^c^	0.128 ± 0.018^b^	83.239
17.6 (N/P ratio ≈ 77)	14.867 ± 2.889^b^	0.257 ± 0.007^a^	0.000
26.47 (N/P ratio ≈ 115)	23.667 ± 2.911^a^	0.258 ± 0.006^a^	14.894*
31.76 (N/P ratio ≈ 138)	24.833 ± 3.156^a^	0.273 ± 0.019^a^	20.739*
*Chlamydomonas debaryana*			
3.52 (N/P ratio ≈ 15)	12.100 ± 1.400^c^	0.111 ± 0.018^c^	74.402
8.82 (N/P ratio ≈ 38)	15.867 ± 2.644^c^	0.163 ± 0.007^b^	47.452
17.6 (N/P ratio ≈ 77)	28.467 ± 1.556^b^	0.211 ± 0.006^a^	0.000
26.47 (N/P ratio ≈ 115)	31.567 ± 2.644^ab^	0.213 ± 0.012^a^	0.891*
31.76 (N/P ratio ≈ 138)	38.267 ± 1.089^a^	0.212 ± 0.002^a^	1.097
K_2_HPO_4_ concentrations (mM)			
*Chlorococcum* sp.			
0.045 (N/P ratio ≈ 384)	9.033 ± 2.778^c^	0.240 ± 0.007^a^	20.362
0.115 (N/P ratio ≈ 153)	11.800 ± 1.667^bc^	0.243 ± 0.009^a^	32.617
0.230 (N/P ratio ≈ 77)	14.867 ± 2.889^b^	0.257 ± 0.007^a^	0.000
0.340 (N/P ratio ≈ 50)	19.133 ± 0.644^ab^	0.239 ± 0.023^a^	15.554
0.410 (N/P ratio ≈ 42)	21.600 ± 2.267^a^	0.259 ± 0.011^a^	4.996*
*Chlamydomonas debaryana*			
0.045 (N/P ratio ≈ 384)	6.133 ± 0.622^d^	0.132 ± 0.011^c^	64.733
0.115 (N/P ratio ≈ 153)	13.333 ± 0.489^c^	0.169 ± 0.007^b^	42.309
0.230 (N/P ratio ≈ 77)	28.467 ± 1.556^b^	0.211 ± 0.006^a^	0.000
0.340 (N/P ratio ≈ 50)	34.133 ± 2.111^ab^	0.208 ± 0.013^a^	6.103
0.410 (N/P ratio ≈ 42)	36.633 ± 1.089^a^	0.205 ± 0.003^ab^	10.217

*Rate of increase in cell density.

Significant difference was determined by One-way ANOVA with post-hoc Tukey Test. Different letters represent statistically significant differences *p* ˂ 0.05.

#### Effect of nitrogen

As shown by the growth curves in Fig. 1c and d, high concentrations of nitrogen favored the growth of both microalgae. Maximum growth was recorded in the control and nitrogen-enriched cultures (17.6, 26.47 and 31.76 mM NaNO_3_). Similarly, the variation in growth rate between the three treatments remained insignificant (0.257–0.273 d^−1^ for *Chlorococcum* sp. and 0.211–0.212 d^−1^ for *Chlamydomonas debaryana*), while chlorophyll a levels were higher in nitrogen-enriched cultures (Table 1). Furthermore, a remarkable reduction in cell growth was observed in both algae in nitrogen-depleted cultures from day 6 in the case of *Chlorococcum* sp. and from day 8 in the case of *Chlamydomonas debaryana*. This early decline observed in both species is probably due to exhaustion of the nitrogen source in the medium. This was manifested by a significant decrease in chlorophyll a content and cell density. However, *Chlorococcum* sp. was more affected by the nitrogen limitation in the medium; the inhibitory rate exceeded 80%, whereas for *Chlamydomonas debaryana,* it did not reach 75% (Table 1). In addition, a sub-decrease in growth rate was observed up to 0.079 d^−1^ for *Chlorococcum* sp. compared with 0.111 d^−1^ for *Chlamydomonas debaryana* (Table 1).

#### Effect of phosphorus

The effect of different phosphorus concentrations on the growth of the two Chlorophyceae studied is shown in Fig. 1e and f. The variation in phosphate concentrations in the medium did not affect cell growth in *Chlorococcum* sp., whose growth dynamics were similar in the five treatments. Its growth rate varied slightly from 0.240 to 0.257 d^−1^, cell densities fluctuated around 4 × 10^6^ cells mL^−1^, and inhibitory rates did not exceed 32.617% (Table 1). Chlorophyll a production was less affected by changes in phosphorus concentrations compared to the different nitrogen treatments. In *Chlamydomonas debaryana*, a growth reduction was observed at the beginning of culture at 0.410 mM K_2_HPO_4_ and after day 9 at 0.340 mM K_2_HPO_4_. This phosphorus depletion significantly reduced growth rates (0.132 d^−1^ and 0.169 d^−1^, respectively) and increased inhibitory rates from 42.309 to 64.733%. In the other treatments, cell growth was maximal, with a growth rate greater than 0.200 d^−1^ and a chlorophyll a content exceeding 28 µg mL^−1^.

### Effect of different NaCl concentrations on hydrogen peroxide and proline concentrations

In both Chlorophyceae, although no significant increase in H_2_O_2_ content was observed at NaCl concentrations below 100 mM, high proline production was induced. At 400 mM NaCl, H_2_O_2_ levels increased by around threefold compared with the control in the case of *Chlorococcum* sp. and 1.5-fold in the case of *Chlamydomonas debaryana*. As for proline, an approximately 30-fold increase compared with the control culture was recorded in the cells of both algae (Table 2).

**Table 2:** Effects of different NaCl concentrations on the H_2_O_2_ and proline contents of *Chlorococcum* sp. and *Chlamydomonas debaryana*.

NaCl concentrations (mM)	H_2_O_2_ (µmol g^−1^ FW)	Proline (µmol g^−1^ DW)
*Chlorococcum* sp.		
0 (control)	11.663 ± 0.321^c^	84.167 ± 4.444^e^
50	11.775 ± 0.107^c^	569.722 ± 7.407^d^
100	16.108 ± 0.535^b^	1021.389 ± 8.148^c^
200	32.386 ± 0.964^a^	1186.944 ± 12.593^b^
400	34.795 ± 2.570^a^	1313.056 ± 10.741^a^
*Chlamydomonas debaryana*		
0 (control)	19.534 ± 0.535^cd^	5.892 ± 0.311^e^
50	17.854 ± 0.612^d^	39.881 ± 0.519^d^
100	21.499 ± 0.446^c^	71.497 ± 0.570^c^
200	26.231 ± 0.459^b^	83.086 ± 0.881^b^
400	30.147 ± 1.249^a^	91.914 ± 0.752^a^

Values indicate means ± standard deviation (n = 3). Significant difference was determined by One-way ANOVA with post-hoc Tukey Test. Different letters represent statistically significant differences *p* ˂ 0.05.

### Effects of different culture conditions on biomass, carbohydrate and lipid production of *Chlorococcum* sp. and *Chlamydomonas debaryana*

#### Biomass

**Figure 2:**
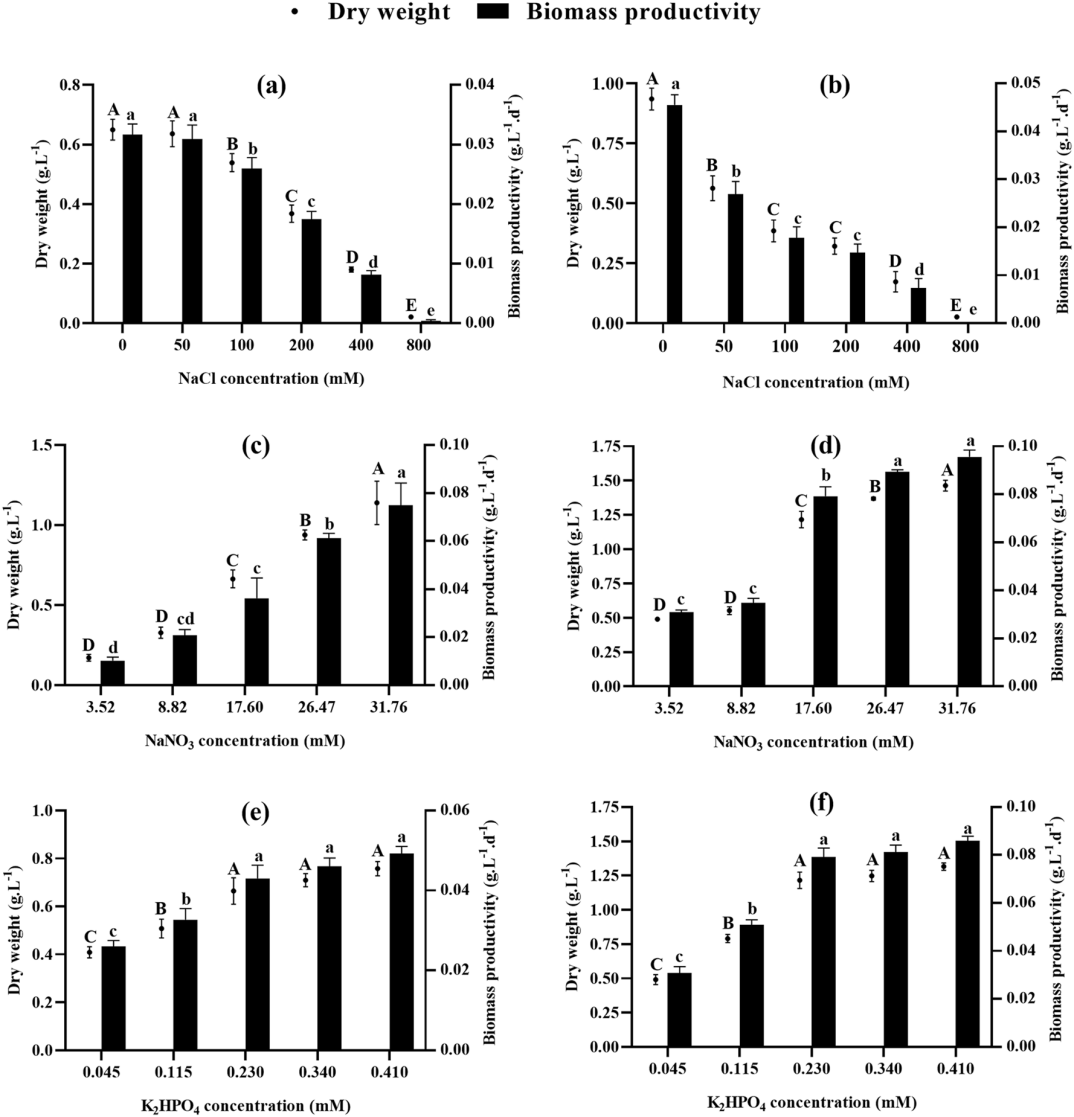
Biomass (dry weight) and biomass productivity of *Chlorococcum* sp. and *Chlamydomonas debaryana* cultivated under different salt, nitrate, and phosphate concentrations. (**a**) Biomass and biomass productivity of *Chlorococcum* sp. cultivated under different salt concentrations. (**b**) Biomass and biomass productivity of *Chlamydomonas debaryana* cultivated under different salt concentrations. (**c**) Biomass and biomass productivity of *Chlorococcum* sp. cultivated under different nitrate concentrations. (**d**) Biomass and biomass productivity of *Chlamydomonas debaryana* cultivated under different nitrate concentrations. (**e**) Biomass and biomass productivity of *Chlorococcum* sp. cultivated under different phosphate concentrations. (**f**) Biomass and biomass productivity of *Chlamydomonas debaryana* cultivated under different phosphate concentrations. (means ± SD). Means followed by different letters are significantly different.

The results in Fig. 2 show that the biomass yield in dry weight and biomass productivity of the two species vary considerably according to culture conditions. Increasing the salinity of the medium leads to a decrease in both parameters in both algae. The lowest values are induced by the highest salinities (800 mM NaCl), i.e., yields of 0.022 and 0.026 g L^−1^ and productivities of 0.0001 and 0.0004 g L^−1^ d^−1^ for *Chlorococcum* sp. and *Chlamydomonas debaryana*, respectively. However, salinities below 200 mM NaCl induced a greater reduction in biomass production in *Chlamydomonas debaryana* (67.69% vs. 44.82% in *Chlorococcum* sp.). Regarding the effect of nutrients, Fig. 2c–f show that low concentrations of nitrogen and phosphorus in the medium have a more marked impact on both algae, leading to reduced yield and biomass productivity. However, medium enrichment in nitrogen favors *Chlorococcum* sp. biomass production, leading to a yield of 1.14 g L^−1^ and a productivity of 0.07 g L^−1^ d^−1^ at 31.76 mM NaNO_3_ (N/P ratio 138), i.e., an increase in productivity of 106% compared to the control (Fig. 2c). On the other hand, nitrogen depletion reduced yield up to 0.17 g L^−1^ and biomass productivity up to 0.01 g L^−1^ d^−1^ at 3.52 mM NaNO_3_ (N/P ratio 15) for the same species. In the case of *Chlamydomonas debaryana*, increasing NaNO_3_ concentrations resulted in a slight increase in yield and biomass productivity (1.46 g L^−1^ and 0.09 g L^−1^ d^−1^ at 31.76 mM NaNO_3_, respectively). Whereas nitrogen-depleted cultures show a significant reduction in both parameters compared to the control culture (*p* ˂ 0.0001) (Fig. 2d). Figure 2e and f show that phosphorus has a less significant effect on biomass production in both algae. Phosphorus-enriched cultures show similar yield and biomass productivity to control cultures. However, biomass production was significantly reduced in phosphorus-depleted cultures. Biomass productivity was reduced from 0.04 g L^−1^ d^−1^ (control) to 0.02 g L^−1^ d^−1^ (0.045 mM K_2_HPO_4_ corresponding to a N/P ratio of 384) for *Chlorococcum* sp. and from 0.08 g L^−1^ d^−1^ (control) to 0.03 g L^−1^ d^−1^ (0.045 mM K_2_HPO_4_ corresponding to a N/P ratio of 384) for *Chlamydomonas debaryana*.

#### Carbohydrates

**Figure 3:**
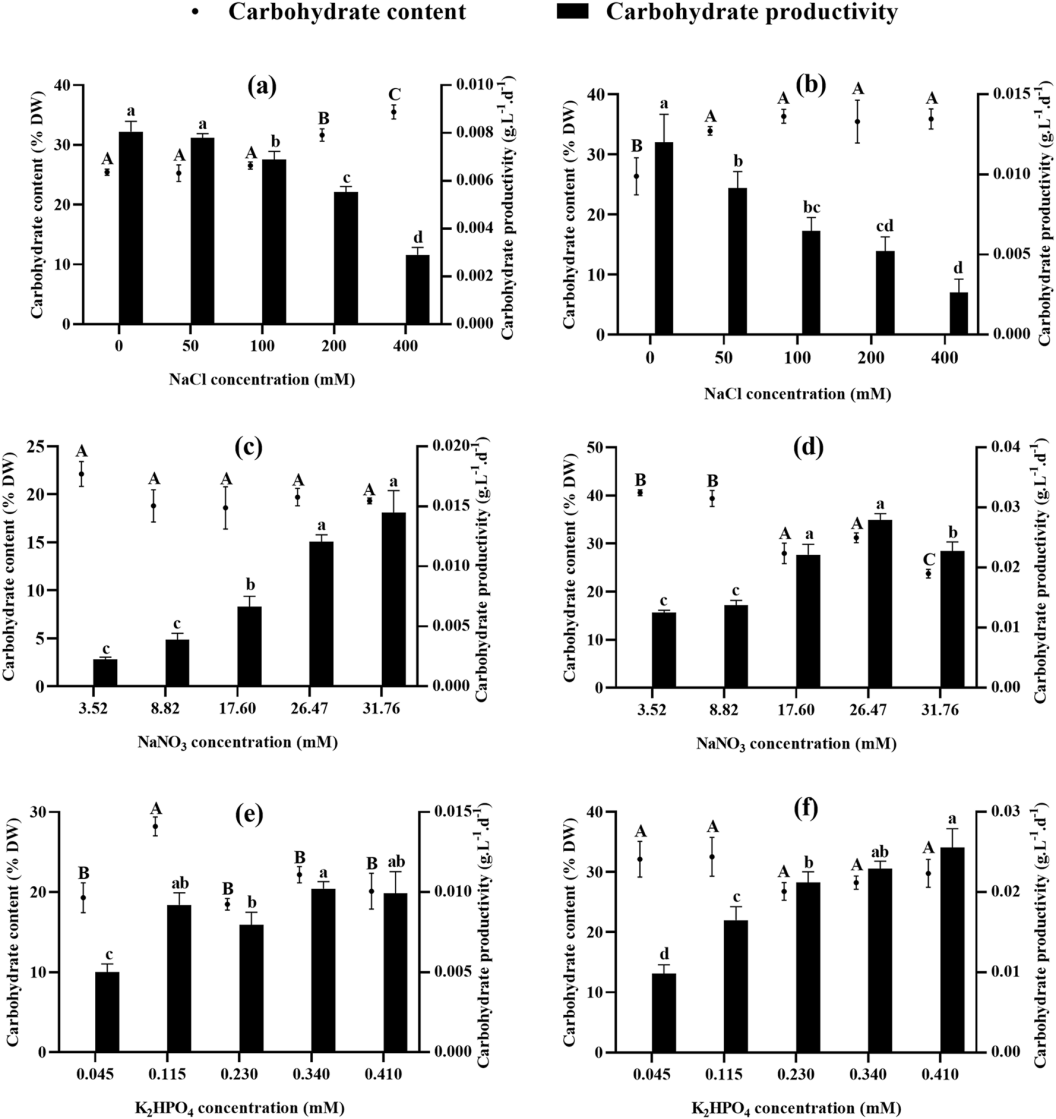
Carbohydrate content and productivity of *Chlorococcum* sp. and *Chlamydomonas debaryana* cultivated under different salt, nitrate, and phosphate concentrations. (**a**) Carbohydrate content and productivity of *Chlorococcum* sp. cultivated under different salt concentrations. (**b**) Carbohydrate content and productivity of *Chlamydomonas debaryana* cultivated under different salt concentrations. (**c**) Carbohydrate content and productivity of *Chlorococcum* sp. cultivated under different nitrate concentrations. (**d**) Carbohydrate content and productivity of *Chlamydomonas debaryana* cultivated under different nitrate concentrations. (**e**) Carbohydrate content and productivity of *Chlorococcum* sp. cultivated under different phosphate concentrations. (**f**) Carbohydrate content and productivity of *Chlamydomonas debaryana* cultivated under different phosphate concentrations. (means ± SD). Means followed by different letters are significantly different.

The results in Fig. 3 show that carbohydrate contents and productivities vary between the two algae studied. However, a decrease in productivity is observed under stress conditions even when carbohydrate content is high, which is due to the decrease in biomass productivity. Salt stress above 100 mM NaCl increases the carbohydrate content and decreases the carbohydrate productivity of both algal species (Fig. 3a,b). However, *Chlamydomonas debaryana* showed no significant variation in carbohydrate content between different salinities, while biomass productivity was significantly reduced. It drops from 0.008 g L^−1^ d^−1^ (control) to 0.003 g L^−1^ d^−1^ (400 mM NaCl), also due to the sharp drop in biomass productivity. As for *Chlorococcum* sp., the effect of increasing salinity is less important, particularly at NaCl concentrations below 200 mM. Although the carbohydrate contents of *Chlorococcum* sp. are similar in the different NaNO_3_ concentrations, its carbohydrate productivity increases considerably in nitrogen-enriched cultures (0.002 g L^−1^ d^−1^ at 31.76 mM) and decreases in nitrogen-depleted cultures (0.014 g L^−1^ d^−1^ at 3.52 mM) (Fig. 3c). However, the greatest effect of nitrogen on *Chlamydomonas debaryana* was observed when the medium was depleted of nitrogen. The lowest concentration of NaNO_3_ (3.52 mM) gave a maximum content of 40.593% and a minimum productivity of 0.012 g L^−1^ d^−1^ (Fig. 3d). Regarding the effect of phosphorus, little variation in carbohydrate content and productivity was observed in the different *Chlorococcum* sp. cultures (Fig. 3e). Nevertheless, the maximum carbohydrate content (28.210%) was recorded at 0.115 mM K_2_HPO_4_, leading to a carbohydrate productivity similar to that of the control and phosphorus-enriched cultures. This is linked to the slight decrease in biomass productivity observed at this concentration. For *Chlamydomonas debaryana*, carbohydrate contents did not vary with changing phosphorus concentrations, while a decrease in productivity was observed at 0.045 and 0.115 mM K_2_HPO_4_ (0.010 and 0.016 g L^−1^ d^−1^, respectively) compared to the control (0.021 g L^−1^ d^−1^) (Fig. 3f).

#### Lipids

**Figure 4:**
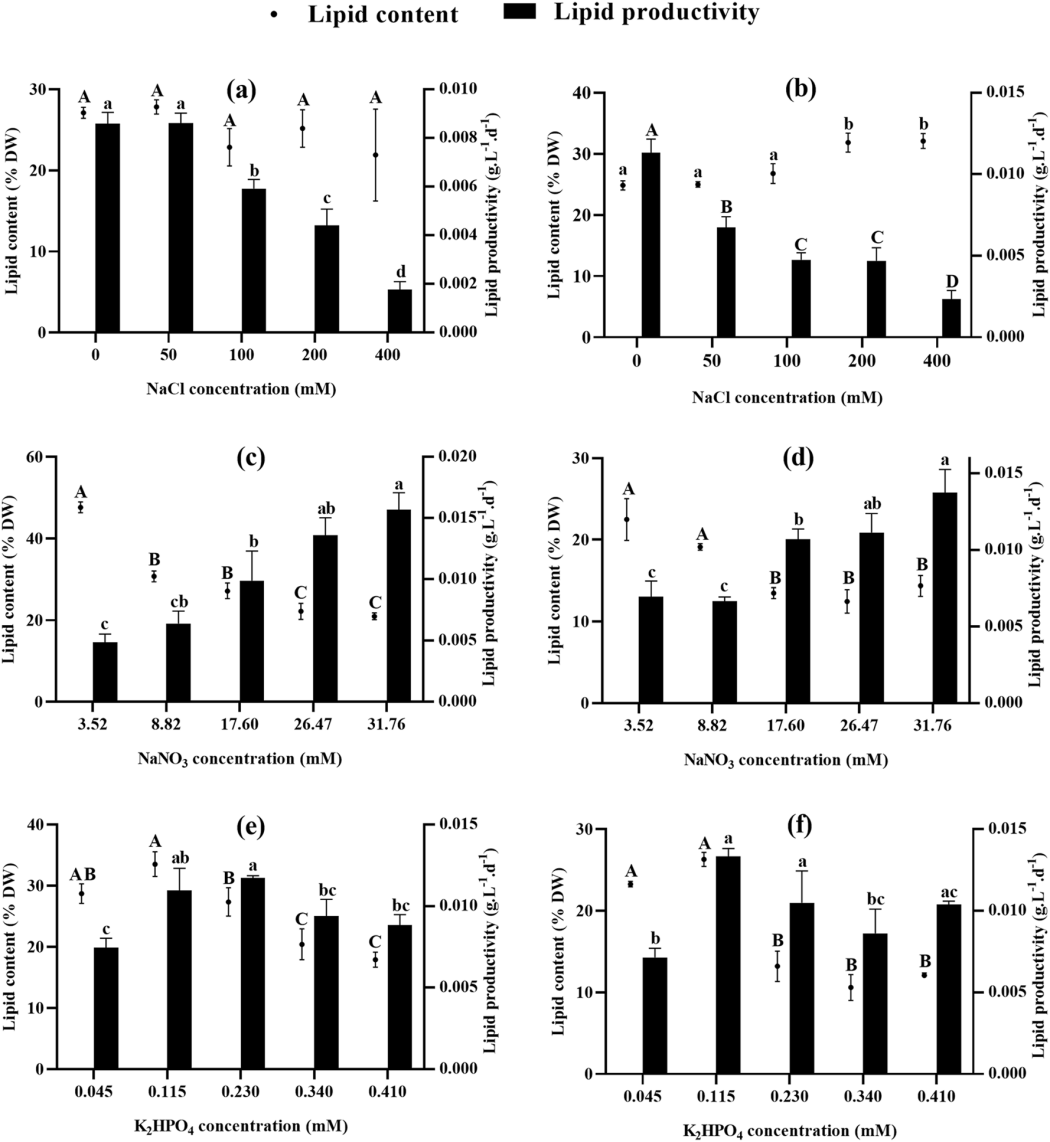
Lipid content and productivity of *Chlorococcum* sp. and *Chlamydomonas debaryana* cultivated under different salt, nitrate, and phosphate concentrations. (**a**) Lipid content and productivity of *Chlorococcum* sp. cultivated under different salt concentrations. (**b**) Lipid content and productivity of *Chlamydomonas debaryana* cultivated under different salt concentrations. (**c**) Lipid content and productivity of *Chlorococcum* sp. cultivated under different nitrate concentrations. (**d**) Lipid content and productivity of *Chlamydomonas debaryana* cultivated under different nitrate concentrations. (**e**) Lipid content and productivity of *Chlorococcum* sp. cultivated under different phosphate concentrations. (**f**) Lipid content and productivity of *Chlamydomonas debaryana* cultivated under different phosphate concentrations. (means ± SD). Means followed by different letters are significantly different.

According to the results in Fig. 4, similar lipid contents are observed at all salinities applied to *Chlorococcum* sp. However, its lipid productivity decreases significantly from 100 mM NaCl, yielding a minimum productivity of 0.002 g L^−1^ d^−1^ at 400 mM. On the other hand, the lipid productivity of *Chlamydomonas debaryana* started to decrease from 50 mM NaCl to reach 0.002 g L^−1^ d^−1^ at 400 mM, with the lowest contents at 200 and 400 mM NaCl (31.833% and 32.096%, respectively, compared to 24.833% for the control) (Fig. 4b). For nitrogen tests, *Chlorococcum* sp. cultures enriched with 31.76 mM NaNO_3_ show maximum lipid productivity (0.016 g L^−1^ d^−1^) despite their low lipid content (20.996%), certainly due to their high biomass productivity. In contrast, nitrogen-depleted cultures show a decrease in lipid productivity and an increase in lipid content, reaching a maximum of 47.636% at 3.52 mM NaNO_3_ (Fig. 4c). Similarly, *Chlamydomonas debaryana* produces high lipid contents under nitrogen-depleted conditions (22.476% at 3.52 mM) with the lowest productivities (0.007 g L^−1^ d^−1^ at 3.52 mM) (Fig. 4d). Although phosphorus-enriched cultures of *Chlorococcum* sp. have higher biomass productivities than phosphorus-depleted cultures, their lipid productivities are similar (around 0.010 g L^−1^ d^−1^) because the lipid contents of phosphorus-enriched cultures are very low (Fig. 4e). *Chlamydomonas debaryana* showed a similar response, with maximum lipid productivity at 0.115 mM K_2_HPO_4_ (0.013 g L^−1^ d^−1^).

### Principal component analysis

**Figure 5:**
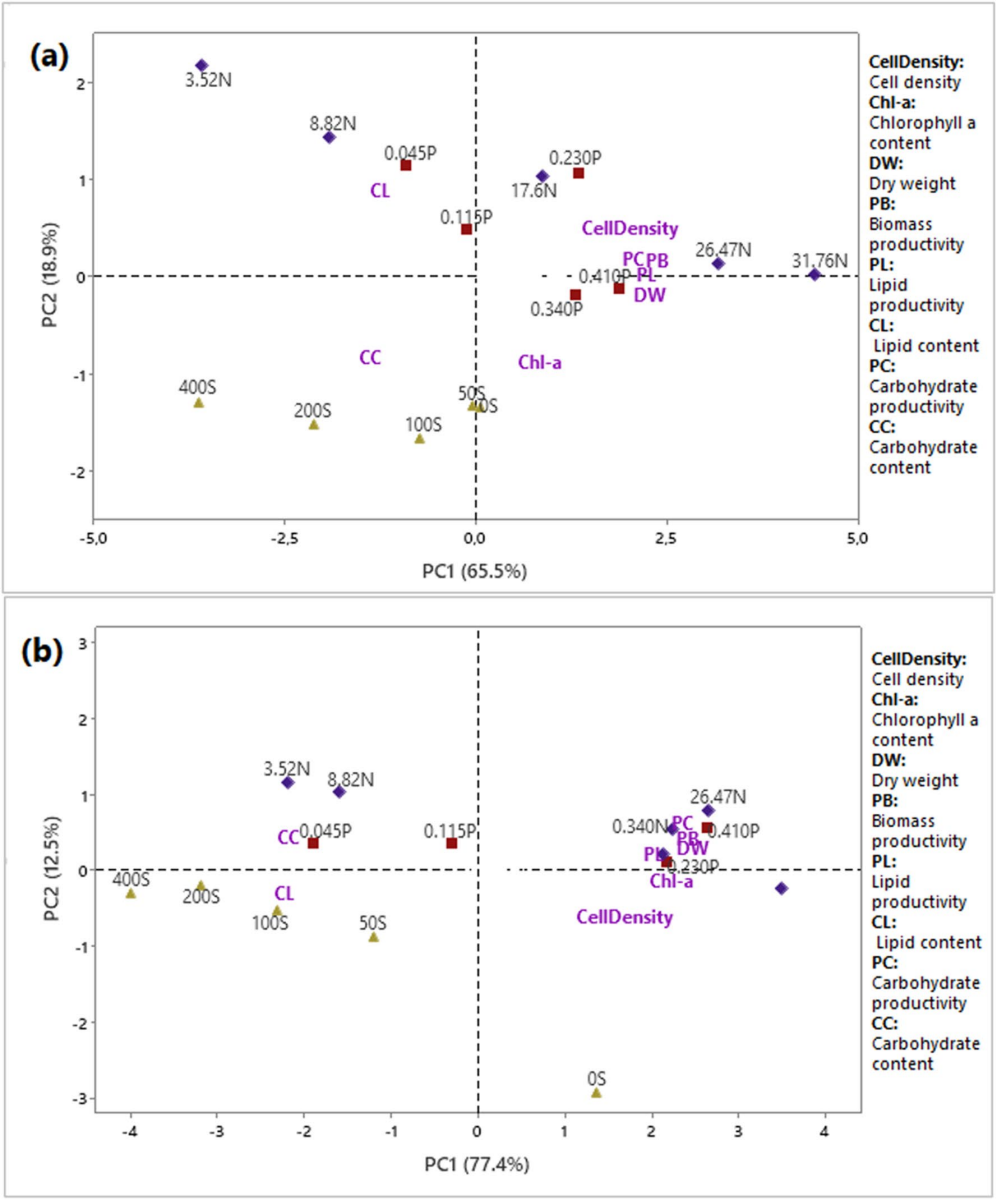
Biplot of the first 2 principal components (PC1 and PC2) of the different parameters studied in *Chlorococcum* sp. (**a**) and *Chlamydomonas debaryana* (**b**). The labels 0S, 50S, 100S, 200S and 400S indicate the different NaCl concentrations; 0.045P, 0.115P, 0.230P, 0.310P and 0.410P indicate the different phosphorus concentrations; 3.52N, 8.82N, 17.6N, 26.47N and 31.76N indicate the different nitrogen concentrations.

Figure 5a and b show the results of principal component analysis performed on parameter data measured in *Chlorococcum* sp. and *Chlamydomonas debaryana*, respectively. The total variation of the data is 84.4% and 89.9%, respectively, distributed over the first two components, PC1 and PC2. It is clear that the productivity of reserve molecules (carbohydrates and lipids) was positively correlated with biomass productivity and favorable growth conditions for both algae, i.e., a nitrogen- and phosphorus-rich environment (N/P ratio between 42 and 138). On the other hand, salt, nitrogen, and phosphorus stress conditions were positively correlated with lipid and carbohydrate content. Nutrient-depleted conditions led to an increase in lipid content, while high salinity led to an accumulation of carbohydrates in *Chlorococcum* sp. An opposite effect was observed in *Chlamydomonas debaryana*.

## Discussion

Environmental conditions such as salinity and nutrients regulate the growth and biochemical composition of microalgae. Understanding the responses of microalgae to changes in such conditions is of great interest for increasing the economic viability of algal biofuels. To this end, lipid or carbohydrate yields need to be maximized while ensuring sufficient algal biomass production, i.e., without inhibiting cell growth. The growth and survival of most freshwater microalgae are reduced at NaCl concentrations above 150 mM^18,19^. However, sensitivity to salt stress remains species-dependent^20^. The results of the present study show that *Chlorococcum* sp. is more tolerant of increasing salinity and can withstand up to 200 mM NaCl. In contrast, the growth of *Chlamydomonas debaryana* was inhibited by more than 50% compared with the control when salinity exceeded 50 mM NaCl (Fig. 1a,b). In the same vein, Talebi et al.^20^ reported a high sensitivity of *Chlorella emersoni* to different levels of NaCl in the medium, showing complete inhibition of growth at salinities above 100 mM. For other species, increasing salinity to a certain level can lead to improved growth. For example, El-Sheekh et al.^21^ reported a 10% increase in *Monoraphidium braunii* growth at NaCl concentrations of 150 mM, above which a significant decrease occurs.

It has been shown that increasing salinity can decrease the amounts of chlorophyll pigments in algal cells, leading to inhibition of photosynthesis and thus reduced growth^22,23^. In the present study, a NaCl concentration of 800 mM resulted in the lowest chlorophyll a content in the two algae studied, reflecting a decrease in photosynthesis and, consequently, an increase in cell death and a decrease in cell density and biomass. Similar results were obtained by^24^, reporting a 93.42% decrease in chlorophyll a content in the microalga *Scenedesmus obliquus* XJ002 at 200 mM NaCl compared to the control. This decrease in the chlorophyll content of microalgae may be due to the negative effect of high salinity on the expression of genes involved in chlorophyll synthesis^23^. However, Sedjati et al.^25^ have reported that photosynthetic activity can be maintained at high salinity by increasing the production of carotenoids, which enhance photon capture and protect the cell. Cell death also results from the accumulation of reactive oxygen species (ROS) such as hydrogen peroxide (H_2_O_2_) in microalgae. These molecules cause oxidative stress, enzyme inactivation, and ionic imbalance in the cells^26,27^. In the present study, an approximately threefold increase in H_2_O_2_ production in *Chlorococcum* sp. and an approximately 1.5-fold increase in *Chlamydomonas debaryana* were induced by increasing the salinity of the medium compared to the control (Table 2). In order to protect cells from ROS-induced damage, microalgae have developed several adaptive strategies, such as the production of osmoprotectants, including proline^20,28^. This amino acid enables the cell to regulate its intracellular osmotic pressure and prevent water loss from the cell. A progressive increase in proline content in all treated cultures as a function of NaCl concentration is clearly observed for both species studied (Table 2). In line with our results, significant H_2_O_2_ accumulation (41.43 μM g^−1^ FW) and high proline production (156.53 μM g^−1^ FW) were reported in *Chlorella vulgaris* YH703 cells at 600 mM NaCl^29^. In addition, proline synthesis can also be affected by nitrogen deprivation in the medium, as it is a nitrogen-based osmoregulator^30,31^. It should be noted that a number of other solutes can be produced by microalgae under stress conditions to counteract stress and enable balanced growth^28^. Furthermore, our results suggest that the two microalgae studied have developed different strategies to mitigate the reactive oxygen species (ROS) generated and to tolerate salt stress. As a result, *Chlorococcum* sp. proved more tolerant of high salinity than *Chlamydomonas debaryana*.

On the other hand, the nutrient composition of the medium, particularly nitrogen and phosphorus, has a considerable effect on microalgae growth^11^. Nitrogen accounts for between 1% and over 10% of microalgae biomass and has a major effect on their growth^32^. In the present study, nitrogen enrichment of the medium had a positive effect on the growth of microalgae, in particular *Chlorococcum* sp. This can be explained by a significant synthesis of nitrogen compounds required for cell division, such as chlorophyll a, leading to high growth rates (Table 1). In a similar vein, Sedjati et al.^25^ have shown that the availability of nitrogenous nutrients enables microalgae to increase their production of proteins and chlorophyll required for growth. In general, microalgae can tolerate low concentrations of nitrogen in the medium, but there is a certain limit below which their growth rate will be affected^33^. In nitrogen-deficient cultures, growth was inhibited by over 80% for *Chlorococcum* sp. and by 47.45–74.40% for *Chlamydomonas debaryana*. This reduction in cell growth resulted in a sharp drop in growth rates (Table 1), chlorophyll a content, and biomass for both species. Similarly, previous studies have reported that reduced nitrate concentration led to a decrease in chlorophyll a content and dry weight in *Chlorella vulgaris*^34^. Lack of nitrogen can have a negative impact on photosynthetic capacity through reduced synthesis of nitrogen compounds, including chlorophyll and proteins, or even their degradation^35,36^. From a molecular point of view, it has been reported by^37^ and^38^ that a lack of nitrogen in the environment results in the down-regulation of proteins involved in chlorophyll biosynthesis and the up-regulation of those involved in chlorophyll degradation. In addition to nitrogen concentration, the form of nitrogen used can also affect microalgae growth^39^. However, nitrate (NO_3_^−^) remains the most commonly used form of inorganic nitrogen for microalgae cultivation^32,33^.

As for phosphorus, which represents 1–5% of algal biomass^32,40^, it is a component of several essential molecules such as nucleic acids, membrane phospholipids and ATP^33,36,41^. Nevertheless, the current study reveals that phosphorus deficiency has a less significant effect on microalgal growth compared to nitrogen deficiency, particularly for *Chlorococcum* sp. The same observation was reported by El-Sheekh et al.^21^ for the microalga *Monoraphidium braunii*. In the case of *Chlamydomonas debaryana*, the effect of varying phosphorus concentrations is greater, with an inhibition rate of 64.72% at 0.045 mM K_2_HPO_4_. This may be explained by a slower metabolism and cell division in this species in the lack of phosphorus. It is probable that the two species studied have different capacities to absorb and store phosphorus, depending on its abundance in the environment. Previous studies have reported that species with high “luxury uptake” can store phosphorus in the form of polyphosphates for use when phosphorus is lacking^40,42,43^.

Furthermore, the ratio of nitrogen to phosphorus is a factor that can limit or favor microalgae growth, as it determines nitrogen or phosphorus deficiency. The optimum N/P ratio remains specific to each algal group or even species^44,45^. The two species studied show good growth in the BG-11 medium, characterized by its high N/P ratio (around 80). This indicates that both algae require a high N/P ratio. Their growth is mainly affected by an N/P ratio below 40, corresponding to the 3.52 and 8.82 mM NaNO_3_ treatments.

In addition, the N/P ratio influences the biochemical composition of microalgae. To adapt to a lack of nitrogen or phosphorus in the environment, each microalgal species develops a specific strategy that enables it to adjust its carbon metabolism and synthesize different macromolecules. However, the majority of microalgae increase the production of reserve molecules when under stress^33,44^. In the present study, increasing salinity and depleting the environment of nitrogen and phosphorus increased the lipid and carbohydrate contents of *Chlorococcum* sp. and *Clamydomonas debaryana* (Figs. 3, 4). However, the same conditions reduced growth rate and biomass productivity, leading to low lipid and carbohydrate productivity. Similar results have been reported for other freshwater microalgae such as *Monoraphidium braunii*^21^ and *Scenedesmus* sp.^46^. An exception was observed at a K_2_HPO_4_ concentration of 0.115 mM; the increase in lipid and carbohydrate content was not accompanied by a significant loss of biomass. As a result, lipid and carbohydrate productivity were similar to the control. At this concentration, lipid productivity of 0.013 g L^−1^ d^−1^ (0.010 g L^−1^ d^−1^ at control) and carbohydrate productivity of 0.009 g L^−1^ d^−1^ (0.008 g L^−1^ d^−1^ at control) were recorded by *Chlorococcum* sp. and 0.010 g L^−1^ d^−1^ lipids (0.012 g L^−1^ d^−1^ control) and 0.016 g L^−1^ d^−1^ (0.021 g L^−1^ d^−1^ control) were observed in *Chlamydomonas debaryana*.

Regarding salinity, no significant effect was observed on lipid production in *Chlorococcum* sp. On the other hand, a substantial increase was induced in *Chlamydomonas debaryana* under 200 and 400 mM NaCl. Similarly, Rai et al.^47^ obtained a maximum lipid production of 26.84% in *Chlorella* sp. at 500 mM NaCl, compared with 14% in the control culture, and Guimarães & França^48^ reported a maximization of lipid accumulation in two microalgae, *Nannochloropsis* sp. and *Pediastrum tetras*, following an increase in salinity. Furthermore, a 37–39% increase in carbohydrate levels was induced in both algae by increasing salinity. A similar effect was reported by^49^, indicating that increasing salinity causes an increase in the carbohydrate content of the Chlorococcum strain studied. It should be noted that although increasing salinity favors lipid and carbohydrate accumulation in the two algae studied, it results in a marked reduction in growth leading to the lowest productivity in terms of biomass, lipids and carbohydrates. In contrast to our results, Teh et al.^50^ observed that a salinity of 15 ppt resulted in the highest lipid content (63.5% of dry weight) in *Chlorella vulgaris* UMT-M1 without compromising biomass productivity.

Although both algae showed a reduction in biomass accompanied by energy storage (negative correlation shown by PCA between biomass and carbohydrate and lipid contents), the form of storage is variable. For *Chlorococcum* sp., the highest lipid contents are obtained at 3.52 mM NaNO_3_ (47.636% DW), while the highest carbohydrate contents are observed at 400 mM NaCl (35.511% DW). Conversely, *Chlamydomonas debaryana* recorded maximum lipid content at 400 mM NaCl (32.096% DW) and maximum carbohydrate content at 3.52 mM NaNO_3_ (40.593% DW). The literature reports that, depending on the type and level of stress, some species accumulate lipids as reserve molecules, others invest in carbohydrate production, while some species increase production of both types of molecules^30^. In addition, microalgae can shift their metabolism from carbohydrate to lipid synthesis depending on the level or duration of stress application, given that the metabolic pathways of both compounds share the same precursors^19^. For example, Chiu et al.^51^ reported that under nitrogen stress, the Chlamydomonas strain studied showed a predominance of carbohydrate production, while the addition of salt stress forced the conversion of starch granules into lipid droplets. In another study, *Chlorella sorokiniana* showed a high starch content during the initial phase of nitrogen depletion, which was replaced by an increase in neutral lipid content during later phases^52^. Elsewhere, Ho et al.^53^ have shown that the commutation from carbohydrate to lipid synthesis in *Chlamydomonas* sp. JSC4 is linked to genetic mechanisms.

Furthermore, it would be more interesting to study the combined effect of nitrogen, phosphorus and salinity because of the interactions these factors can have with regard to biomolecule synthesis. In this context, Singh et al.^54^ showed a significant increase in lipid productivity under a combined nutrient stress of nitrogen, phosphorus, and iron in *Ankistrodesmus falcatus* KJ671624. In addition, the study needs to be completed by culturing *Chlorococcum* sp. and *Chlamydomonas debaryana* under the determined lipid and carbohydrate accumulation conditions, while seeking to maintain sufficient biomass production. In this respect, it would be interesting to optimize medium composition and culture conditions using the design of experiments method, or to proceed with culture in two stages^33^: a first stage under optimal conditions to ensure high biomass productivity, and a second stage of culture under stress conditions to maximize lipid or carbohydrate production.

## Materials and methods

### Microalgae and culture conditions

**Figure 6:**
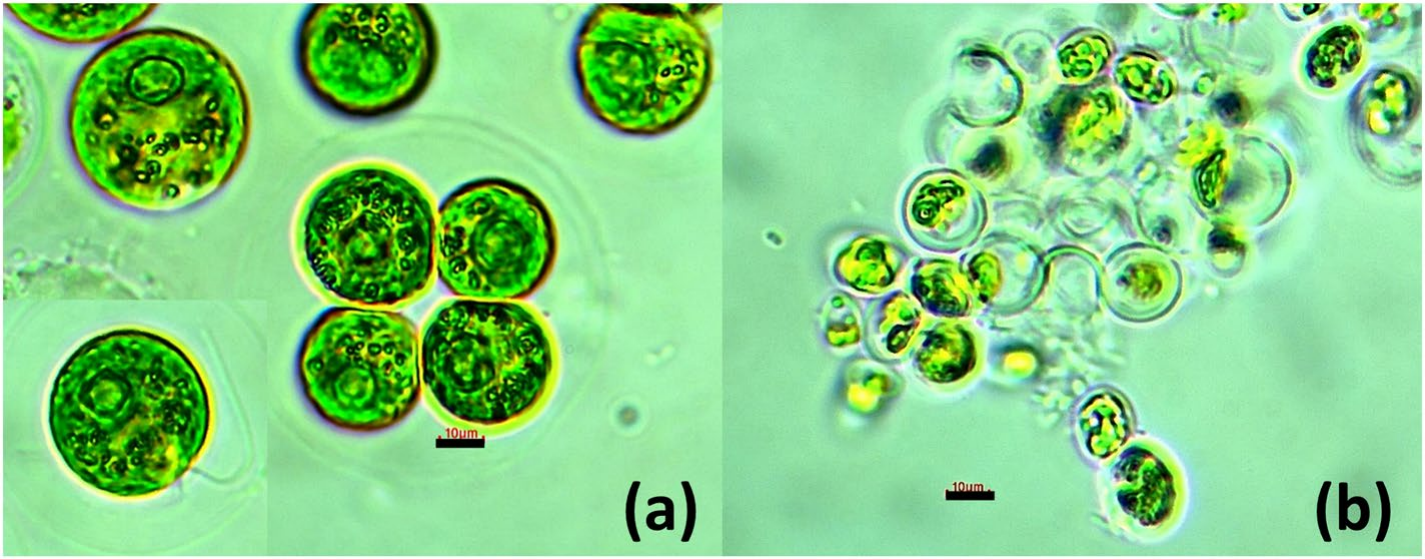
Light microscopy image of (**a**) *Chlamydomonas debaryana* and (**b**) *Chlorococcum* sp. Scale bar represents 10 µm.

The two microalgae studied in this work were isolated from two freshwater ecosystems in the city of Fez (Morocco) using the Bold Basal Medium (BBM) plating technique. Successive subculturing enabled the establishment of pure cultures and microscopic examination allowed the morphological identification of the two strains according to^55^ as *Chlamydomonas debaryana* (Class: Chlorophyceae; Order: Chlamydomonadales; Family: Chlamydomonadaceae) isolated from the artificial reservoir El Gaada (34° 0′ 58″ N 4° 57′ 1″ W) and *Chlorococcum* sp. (Class: Chlorophyceae; Order: Chlamydomonadales; Family: Chlorococcaceae) isolated from the Oued Fez River (34° 02′ 24.1″ N 5° 03′ 42.1″ W). The two Chlorophyceae were maintained in BBM medium in a culture chamber at a temperature of 25 °C under continuous light (40 µmol photons m^−2^ s^−1^). Figure 6 shows micrographs of the two isolates.

The two isolates were then grown in BG-11 medium until the exponential phase to prepare the inoculum, under continuous light (40 µmol photons m^−2^ s^−1^) at 30 °C and pH 7. The BG-11 medium consists of: NaNO_3_ (1.5 g L^−1^); K_2_HPO_4_ (0.04 g L^−1^); MgSO_4_. 7H_2_O (0.075 g L^−1^); CaCl_2_. 2H_2_O (0.036 g L^−1^); Ferric ammonium citrate (0.006 g L^−1^); Na_2_EDTA (0.001 g L^−1^); Na_2_CO_3_ (0.02 g L^−1^); H_3_BO_3_ (2.86 g L^−1^); MnCl_2_. 4H_2_O (1.81 g L^−1^); ZnSO_4_. 7H_2_O (0.222 g L^−1^); Na_2_MoO_4_. 2H_2_O (0.39 g L^−1^); CuSO_4_. 5H_2_O (0.079 g L^−1^); Co (NO_3_)_2_. 6H_2_O (0.0494 g L^−1^).

100 mL of algal inoculums were centrifuged for 5 min at 4400 × *g* and 4 °C and the harvest algal cells were washed three times with distilled water and resuspended in 1 L fresh medium according to three series of experiments.

To study the effect of salinity, the two microalgae were first grown in salinities of up to 2000 mM NaCl based on literature research relating to the effect of salinity on freshwater microalgae. These tests showed that NaCl concentrations above 800 mM are lethal for both microalgae. Consequently, the tests were carried out by cultivating the two algae in BG-11 medium at different initial NaCl concentrations of 0 mM (control), 50 mM, 100 mM, 200 mM, 400 mM and 800 mM.

To experiment the effect of nitrogen and phosphorus concentrations, cultures were carried out in BG-11 medium containing different concentrations of NaNO_3_ and K_2_HPO_4_. Two low concentrations (20 and 50% of control BG-11 concentration) were used to test the effect of nutrient deficiency and two high concentrations (150 and 180% of control BG-11 concentration) were used to test the effect of nutrient excess:

Nitrogen experiments: cultures were carried out in BG-11 medium containing 3.52 mM (20% N depletion), 8.82 mM (50% N depletion), 17.6 mM (control), 26.47 mM (150% N enrichment) and 31.76 mM (180% N enrichment), the initial phosphorus concentration being 0.23 mM for all experiments (Table 3).

**Table 3:** Initial NaNO_3_ and K_2_HPO_4_ concentrations and N/P ratios used in nutrient effect experiments.

	Nutrient depletion		Control	Nutrient enrichment	
Initial NaNO_3_ concentration (mM)^a^	3.52	8.82	17.60	26.47	31.76
Pourcentage of BG-11 concentration	20%	50%	100%	150%	180%
N/P ratio	15.37	38.42	76.85	115.27	138.32
Initial K_2_HPO_4_ concentration (mM)^b^	0.045	0.115	0.230	0.320	0.410
Pourcentage of BG-11 concentration	20%	50%	100%	150%	180%
N/P ratio	384.23	153.69	76.85	51.23	42.69

^a^The initial K_2_HPO_4_ concentration was 0.230 mM.

^b^The initial NaNO_3_ concentration was 17.60 mM.

Phosphorus experiments: cultures were carried out in BG-11 medium containing different concentrations of K_2_HPO_4_; 0.045 mM (20% P depletion), 0.115 mM (50% P depletion), 0.23 mM (control), 0.32 mM (150% P enrichment) and 0.41 mM (180% P enrichment), the initial nitrogen concentration being 17.6 mM for all experiments (Table 3).

Three replicates of each experiments were carried out in batch culture in 2 L Erlenmeyer flasks at 30 °C and pH 7, under continuous light (40 µmol photons m^−2^ s^−1^) for a period of 15–20 days and the cultures were manually shaken twice a day to maintain cells suspension.

### Analytical methods

#### Cell density and growth rate

To monitor culture growth, cell density was daily estimated by measuring absorbance at 680 nm using a spectrophotometer (UV-2005). The values were then converted into cell densities using the correlation curve between cell density and culture absorbance. The specific growth rate (µ) was calculated from the cell density using the following Eq. (1):

1 $$\upmu \left( {{\text{d}}^{{ - {1}}} } \right) = ln{{\left( {\frac{Xt}{{X0}}} \right)} \mathord{\left/ {\vphantom {{\left( {\frac{Xt}{{X0}}} \right)} {\left( {t - t0} \right)}}} \right. \kern-0pt} {\left( {t - t0} \right)}}$$

where X_t_ and X_0_ are defined as the cell density at time t (15 days) and t_0_ (0 days), respectively.

#### Inhibitory rate

The inhibitory rate (IR) of the two microalgae was determined for each treatment according to the following Eq. (2)^56^:

2 $${\text{IR}} = ({\text{X}}0 - {\text{Xt}}/{\text{X}}0) \times 100$$

where X_0_ and X_t_ represent the cell densities of the control and treated cultures at day 15, respectively.

#### Estimation of chlorophyll a content

Chlorophyll a was extracted according to^57^ using 90% acetone. The samples were incubated for 24 h at 4 °C and the chlorophyll a content was measured spectrophotometrically according to SCOR-UNESCO^58^ Eq. (3):

3 $${\text{Chl}}.{\text{a }}\left( {\upmu {\text{g}}\;{\text{L}}^{{ - {1}}} } \right) = \frac{{{\text{v}}\left( {11,64{\text{*OD}}663 - 2,16{\text{*OD}}645 - 0,1{\text{*OD}}630} \right)}}{{{\text{VL}}}}$$

where v: volume of acetone extract in mL, V: sample volume in mL, L: optical path of the cell in cm, OD_xxx_: optical density at xxx nm.

#### Biomass determination

A known volume culture was taken at the end of the experiments and centrifuged at 4400 × *g* at 4 °C for 5 min. The harvested biomass was then rinsed three times with distilled water to remove salts. After centrifugation, it was dried at 60 °C to a constant weight^57^. Biomass concentration is given as g dry weight L^−1^ and used to calculate biomass productivity (PB) using the Eq. (4):

4 $${\text{PB}}\left( {{\text{g}}\,{\text{L}}^{{ - {1}}} \,{\text{d}}^{{ - {1}}} } \right) = \frac{Xt - X0}{{t - t0}}$$

where X_0_ and X_t_ are the initial (time t_0_) and the final (time t) biomass concentration, respectively.

#### Determination of total lipid content

Lipids were extracted using the method of Folch^59^ at the end of culture. The dried biomass was homogenized with chloroform/methanol (2:1 v/v). The mixture was then stirred for 15 min at room temperature and centrifuged for five minutes at 4400 × *g* and 4 °C. 0.2 volumes of distilled water were added to the supernatant to separate the two phases. The upper alcoholic phase was eliminated while the lower chloroformic phase, containing the lipids, was evaporated on a rotary evaporator. The lipids were then determined by colorimetry^60^ using palmitic acid as the standard. Results were expressed as % dry weight and then used to calculate lipid productivity (PL) according to the Eq. (5):

5 $${\text{PL}}\left( {{\text{g}}\;{\text{L}}^{{ - {1}}} \;{\text{d}}^{{ - {1}}} } \right) = {\text{Biomass productivity}} \times {\text{Lipid content}}$$

#### Determination of total carbohydrate content

Carbohydrates were estimated using the Dubois method adopted by Alaoui^61^ on the final day of culture. Dried biomass was reacted with 1 mL of phenol (5%) and 5 mL of sulphuric acid then incubated for 20 min at 100 °C in a water bath. The absorbance was measured at 490 nm and concentrations were determined from a calibration curve performed at different glucose concentrations. Results are expressed as % dry weight and then used to calculate carbohydrate productivity (PC) according to the Eq. (6):

6 $${\text{PC}}\left( {{\text{g}}\;{\text{L}}^{{ - {1}}} \;{\text{d}}^{{ - {1}}} } \right) = {\text{Biomass productivity}} \times {\text{Carbohydrate content}}$$

#### Determination of stress biomarkers

Salt stress was assessed by determining the levels of proline and hydrogen peroxide (H_2_O_2_) in the cultures at different NaCl concentrations. The proline content of the dry biomass was determined colorimetrically based on its reaction with ninhydrin^62^ and using L-proline as a standard. Variations in H_2_O_2_ content were assessed using the method of^63^. Fresh algal biomass was homogenized with a 0.1% w/v TCA solution. After centrifuging the mixture at 5000 rpm for 5 min, 0.5 mL of the supernatant was mixed with 1 mL of 1 M potassium iodide (KI) and 0.5 mL of 10 mM phosphate buffer (pH 7.0). The H_2_O_2_ content was expressed as µmol.g^-1^ fresh weight after reading the absorbance at 390 nm.

### Statistical analysis

GraphPad Prism 8.0.1 package for Windows version 244 was used for all statistical analyses. Two-way ANOVA followed by the Tukey post-hoc test were used to compare differences in the means (*p* < 0.05). The means ± standard deviation were used to represent the results, with different letters denoting significant differences.

To create a biplot of the studied parameters, principal component analysis (PCA) was carried out under Minitab 21 version 21.4.2, using KMO and Bartlett's test as well as Oblimin with Kaiser Normalization in Rotation Method.

## Conclusion

The physiological and biochemical responses to the different culture conditions studied were variable in the two algae studied. While excessive amounts of nitrogen and phosphorus had no major effects on the growth, limiting these two nutrients and increasing the salinity had considerable negative effects. *Chlorococcum* sp. showed greater adaptation to increased salinity and phosphorus depletion than *Chlamydomonas debaryana*.

In general, the stress conditions applied (increased salinity and nitrogen and phosphorus limitation) induced a loss of biomass accompanied by energy storage in the form of lipids or carbohydrates, and as a result, low lipid and carbohydrate productivities are recorded. Nevertheless, at 0.115 mM K_2_HPO_4_, the increase in lipid and carbohydrate content is not accompanied by a significant loss of biomass. Consequently, lipid and carbohydrate productivities are similar to the control. For *Chlorococcum* sp., the maximum carbohydrate production was obtained from 200 mM NaCl (35.51 ± 0.83% DW) while the maximum lipid production was obtained at 3.52 mM NaNO_3_ (47.64 ± 0.88% DW). For *Chlamydomonas debaryana*, carbohydrate production was essentially increased under nitrogen and phosphorus stress with the highest content at less than 8.82 mM NaNO_3_ (40.59 ± 0.46% DW), while maximum lipid production was obtained under salt stress greater than 200 mM NaCl (32.10 ± 0.91% DW).

The carbohydrate-rich *Chlamydomonas debaryana* can be used for bioethanol production. While the lipid-accumulating *Chlorococcum* sp. could be a feedstock for biodiesel. The above conditions are then selected to maximize lipid and carbohydrate production from these two algae. They will be used as a starting point for optimizing biofuel production.

## Data Availability

The datasets used and/or analyzed during the current study are available from the corresponding author on reasonable request.

## References

[1] Bajpai P, Bajpai P (2019). Fuel potential of third generation biofuels. Third Generation Biofuels.

[2] Ganesan R (2020). A review on prospective production of biofuel from microalgae. Biotechnol. Rep..

[3] Mutanda T (2011). Bioprospecting for hyper-lipid producing microalgal strains for sustainable biofuel production. Bioresour. Technol..

[4] Markou G, Nerantzis E (2013). Microalgae for high-value compounds and biofuels production: A review with focus on cultivation under stress conditions. Biotechnol. Adv..

[5] Magierek E, Krzemińska I (2018). Effect of stress conditions on improvement of lipid and carbohydrate accumulation under photoautotrophic cultivation of Chlorophyta. Phycologia.

[6] Jazzar S, Berrejeb N, Messaoud C, Marzouki MN, Smaali I (2016). Growth parameters, photosynthetic performance, and biochemical characterization of newly isolated green microalgae in response to culture condition variations. Appl. Biochem. Biotechnol..

[7] Krichen E, Rapaport A, Le Floc’h E, Fouilland E (2019). Demonstration of facilitation between microalgae to face environmental stress. Sci. Rep..

[8] Briddon CL (2022). The combined impact of low temperatures and shifting phosphorus availability on the competitive ability of cyanobacteria. Sci. Rep..

[9] Bibi F, Jamal A, Huang Z, Urynowicz M, Ishtiaq Ali M (2022). Advancement and role of abiotic stresses in microalgae biorefinery with a focus on lipid production. Fuel.

[10] Chu F-F (2013). Phosphorus plays an important role in enhancing biodiesel productivity of *Chlorella vulgaris* under nitrogen deficiency. Bioresour. Technol..

[11] Yaakob MA, Mohamed RMSR, Al-Gheethi A, Aswathnarayana Gokare R, Ambati RR (2021). Influence of nitrogen and phosphorus on microalgal growth, biomass, lipid, and fatty acid production: An overview. Cells.

[12] Pandit PR, Fulekar MH, Karuna MSL (2017). Effect of salinity stress on growth, lipid productivity, fatty acid composition, and biodiesel properties in *Acutodesmus obliquus* and *Chlorella vulgaris*. Environ. Sci. Pollut. Res..

[13] Hassi M, Mohamed A, Ouaddi O, Oukarroum A (2020). A review of Moroccan microalgae and their exploitation fields. IOSR.

[14] Maadane A (2015). Antioxidant activity of some Moroccan marine microalgae: Pufa profiles, carotenoids and phenolic content. J. Biotechnol..

[15] El Arroussi H (2017). Screening of marine microalgae strains from Moroccan coasts for biodiesel production. Renew. Energy.

[16] Idrissi AA, Mohamed B, Mohammed AM, Lotfi A (2016). Growth performance and biochemical composition of nineteen microalgae collected from different Moroccan reservoirs. Mediterr. Mar. Sci..

[17] Youssef M, Brakez Z, Yassine E, Lhoucine B (2020). Investigation of lipid production and fatty acid composition in some native microalgae from Agadir region in Morocco. Afr. J. Biotechnol..

[18] Shetty P, Gitau M, Maróti G (2019). Salinity stress responses and adaptation mechanisms in eukaryotic green microalgae. Cells.

[19] Zhang L (2018). Salinity-induced cellular cross-talk in carbon partitioning reveals starch-to-lipid biosynthesis switching in low-starch freshwater algae. Bioresour. Technol..

[20] Talebi AF, Tabatabaei M, Mohtashami SK, Tohidfar M, Moradi F (2013). Comparative salt stress study on intracellular ion concentration in marine and salt-adapted freshwater strains of microalgae. Not. Sci. Biol..

[21] El-Sheekh MM, Galal HR, Mousa ASH, Farghl AAM (2024). Impact of macronutrients and salinity stress on biomass and biochemical constituents in *Monoraphidium braunii* to enhance biodiesel production. Sci. Rep..

[22] Bartolomé MC, D’ors A, Sánchez-Fortún S (2009). Toxic effects induced by salt stress on selected freshwater prokaryotic and eukaryotic microalgal species. Ecotoxicology.

[23] Li S (2022). Mechanism study on the regulation of metabolite flux for producing promising bioactive substances in microalgae *Desmodesmus* sp. YT through salinity stress. Algal Res..

[24] Ji X (2018). The effect of NaCl stress on photosynthetic efficiency and lipid production in freshwater microalga—*Scenedesmus obliquus* XJ002. Sci. Total Environ..

[25] Sedjati S (2019). Chlorophyll and carotenoid content of *Dunaliella salina* at various salinity stress and harvesting time. IOP Conf. Ser. Earth Environ. Sci..

[26] Sun X-M (2018). Influence of oxygen on the biosynthesis of polyunsaturated fatty acids in microalgae. Bioresour. Technol..

[27] Chen H, Wang Q (2021). Regulatory mechanisms of lipid biosynthesis in microalgae. Biol. Rev. Camb. Philos. Soc..

[28] Hamed SM, Selim S, Klöck G, AbdElgawad H (2017). Sensitivity of two green microalgae to copper stress: Growth, oxidative and antioxidants analyses. Ecotoxicol. Environ. Saf..

[29] Yun C-J, Hwang K-O, Han S-S, Ri H-G (2019). The effect of salinity stress on the biofuel production potential of freshwater microalgae *Chlorella vulgaris* YH703. Biomass Bioenergy.

[30] Pancha I (2014). Nitrogen stress triggered biochemical and morphological changes in the microalgae *Scenedesmus* sp. CCNM 1077. Bioresour. Technol..

[31] von Alvensleben N, Magnusson M, Heimann K (2016). Salinity tolerance of four freshwater microalgal species and the effects of salinity and nutrient limitation on biochemical profiles. J. Appl. Phycol..

[32] Grobbelaar J, Richmond A (2007). Algal nutrition: Mineral nutrition. Handbook of Microalgal Culture: Biotechnology and Applied Phycology.

[33] Markou G, Vandamme D, Muylaert K (2014). Microalgal and cyanobacterial cultivation: The supply of nutrients. Water Res..

[34] Liu T (2022). Biochemical and morphological changes triggered by nitrogen stress in the oleaginous microalga *Chlorella vulgaris*. Microorganisms.

[35] Li T (2016). Morphology, growth, biochemical composition and photosynthetic performance of *Chlorella vulgaris* (Trebouxiophyceae) under low and high nitrogen supplies. Algal Res..

[36] da Silva Ferreira V, Sant’Anna C (2017). Impact of culture conditions on the chlorophyll content of microalgae for biotechnological applications. World J. Microbiol. Biotechnol..

[37] Li L, Zhang L, Liu J (2021). Proteomic analysis of hydrogen production in *Chlorella pyrenoidosa* under nitrogen deprivation. Algal Res..

[38] Rai V, Muthuraj M, Gandhi MN, Das D, Srivastava S (2017). Real-time iTRAQ-based proteome profiling revealed the central metabolism involved in nitrogen starvation induced lipid accumulation in microalgae. Sci. Rep..

[39] Fatini MA, Basri EM, Maznah WOW (2021). Effect of different nitrogen sources on cell growth and biochemical compositions of *Chlorococcum* sp. cultivated under laboratory conditions. IOP Conf. Ser. Earth Environ. Sci..

[40] Solovchenko A (2019). Phosphorus starvation and luxury uptake in green microalgae revisited. Algal Res..

[41] Liang M-H, Qv X-Y, Chen H, Wang Q, Jiang J-G (2017). Effects of salt concentrations and nitrogen and phosphorus starvations on neutral lipid contents in the green microalga *Dunaliella tertiolecta*. J. Agric. Food Chem..

[42] Procházková G, Brányiková I, Zachleder V, Brányik T (2014). Effect of nutrient supply status on biomass composition of eukaryotic green microalgae. J. Appl. Phycol..

[43] Solovchenko AE (2019). Luxury phosphorus uptake in microalgae. J. Appl. Phycol..

[44] Maltsev Y, Kulikovskiy M, Maltseva S (2023). Nitrogen and phosphorus stress as a tool to induce lipid production in microalgae. Microb. Cell Factories.

[45] Thrane J-E, Hessen DO, Andersen T (2017). Plasticity in algal stoichiometry: Experimental evidence of a temperature-induced shift in optimal supply N:P ratio. Limnol. Oceanogr..

[46] Xin L, Hong-ying H, Ke G, Ying-xue S (2010). Effects of different nitrogen and phosphorus concentrations on the growth, nutrient uptake, and lipid accumulation of a freshwater microalga *Scenedesmus* sp. Bioresour. Technol..

[47] Rai MP, Gautom T, Sharma N (2015). Effect of salinity, pH, light intensity on growth and lipid production of microalgae for bioenergy application. OnLine J. Biol. Sci..

[48] Guimarães BS, França KB (2021). Statistical study of growth kinetics and lipid content of microalgae grown in brackish waters for bioenergetic purposes. Rev. Ambiente Água.

[49] Kirrolia A, Bishnoi N, Singh R (2012). Effect of shaking, incubation temperature, salinity and media composition on growth traits of green microalgae *Chlorococcum* sp.. J. Algal Biomass Util..

[50] Teh KY (2021). Lipid accumulation patterns and role of different fatty acid types towards mitigating salinity fluctuations in *Chlorella vulgaris*. Sci. Rep..

[51] Chiu L, Ho S-H, Shimada R, Ren N-Q, Ozawa T (2017). Rapid in vivo lipid/carbohydrate quantification of single microalgal cell by Raman spectral imaging to reveal salinity-induced starch-to-lipid shift. Biotechnol. Biofuels.

[52] Klok AJ, Lamers PP, Martens DE, Draaisma RB, Wijffels RH (2014). Edible oils from microalgae: Insights in TAG accumulation. Trends Biotechnol..

[53] Ho S-H (2017). Dynamic metabolic profiling together with transcription analysis reveals salinity-induced starch-to-lipid biosynthesis in alga *Chlamydomonas* sp. JSC4. Sci. Rep..

[54] Singh P, Guldhe A, Kumari S, Rawat I, Bux F (2015). Investigation of combined effect of nitrogen, phosphorus and iron on lipid productivity of microalgae *Ankistrodesmus falcatus* KJ671624 using response surface methodology. Biochem. Eng. J..

[55] Wehr JD, Sheath RG, Kociolek JP (2015). Freshwater Algae of North America: Ecology and Classification.

[56] Singh R, Upadhyay AK, Chandra P, Singh DP (2018). Sodium chloride incites reactive oxygen species in green algae *Chlorococcum humicola* and *Chlorella vulgaris*: Implication on lipid synthesis, mineral nutrients and antioxidant system. Bioresour. Technol..

[57] Chng LM, Lee KT, Chan DCJ (2017). Evaluation on microalgae biomass for bioethanol production. IOP Conf. Ser. Mater. Sci. Eng..

[58] Jeffrey SW, Mantoura RFC, Wright SW (1997). Phytoplankton Pigments in Oceanography: Guidelines to Modern Methods.

[59] Folch J, Lees M, Sloane Stanley GH (1957). A simple method for the isolation and purification of total lipides from animal tissues. J. Biol. Chem..

[60] Amenta J (1964). A rapid method for quantification of lipids separated by thin-layer chromatopraphy. J. Lipid Res..

[61] Alaoui, M. M. Dynamique des populations et évolution métabolique du phytoplancton dans un lac eutrophe (Lac Aydat, PUY de DOME, France). Université Blaise Pascal (Clermont-Ferrand II) U.F.R. de la Recherche Scientifique et Technique. *Imp Sciences 63177 Aubiere CEDEX* (1985).

[62] Bates LS, Waldren RP, Teare ID (1973). Rapid determination of free proline for water-stress studies. Plant Soil.

[63] Velikova V, Yordanov I, Edreva A (2000). Oxidative stress and some antioxidant systems in acid rain-treated bean plants. Plant Sci..

